# Gout is associated with a higher risk of chronic renal disease in older adults: a retrospective cohort study of U.S. Medicare population

**DOI:** 10.1186/s12882-019-1274-5

**Published:** 2019-03-15

**Authors:** Jasvinder A. Singh, John D. Cleveland

**Affiliations:** 1 Medicine Service, VA Medical Center, 510, 20th street South, FOT 805B, Birmingham, AL 35233 USA; 2 Department of Medicine at School of Medicine, University of Alabama at Birmingham, 1720 Second Ave. South, Birmingham, AL 35294-0022 USA; 3 Division of Epidemiology at School of Public Health, University of Alabama at Birmingham, 1720 Second Ave. South, Birmingham, AL 35294-0022 USA; 4 University of Alabama at Birmingham, Faculty Office Tower 805B, 510 20th Street S, Birmingham, AL 35294-0022 USA

**Keywords:** Chronic kidney disease, Gout, Older adults, Elderly, Risk

## Abstract

**Background:**

Hyperuricemia and gout have been linked to chronic kidney disease (CKD)**.** Whether the increased risk of CKD in gout is due to shared risk factors such as hypertension, diabetes or heart disease, or due to gout itself is not known. Studies in older adults, who tend to have a high incidence of CKD, are limited. Our objective was to assess whether gout was associated with incident CKD in older adults.

**Methods:**

Using the 5% random sample of Medicare claims, we assessed whether gout is associated with higher risk of incident (new) CKD in adults 65 years or older, using multivariable-adjusted Cox regression analyses, adjusting for demographics (age, gender, race), medical comorbidity and common medications. We calculated hazard ratios (HR) and 95% confidence interval (CI). Sensitivity analyses varied comorbidity variable (models 2, 3), or limited CKD to the most specific codes.

**Results:**

Of the 1,699,613 eligible people, 168,065 developed incident CKD; 150,162 people without gout and 17,903 people with gout. Respective crude incidence rates were 15.6 vs. 78.1 per 1000 person-years. We found that gout was associated with a higher risk of incident CKD in multivariable-adjusted analyses, HR was 3.05 (95% CI, 2.99, 3.10), with minimal attenuation in sensitivity analyses, with HR 2.96 (95% CI, 2.91, 3.01) (model 2, categorical Charlson-Romano) and 2.59 (95% CI, 2.54, 2.63) (model 3, individual Charlson-Romano comorbidities plus hypertension, heart disease, obesity, coronary artery disease). Sensitivity analyses that limited the CKD diagnostic codes to more specific codes, confirmed findings from the main models with respective HRs of 3.10 (95% CI, 3.05, 3.15; Model 1), 3.03 (95% CI, 2.97, 3.08; Model 2) and 2.60 (95% CI, 2.56, 2.65; Model 3).

**Conclusion:**

Gout was associated with a 3-fold higher risk of CKD, confirmed in multiple sensitivity analyses. Future studies should provide insights into underlying mechanisms that are responsible for an increased CKD risk in gout.

## Introduction

One in seven Americans adults, or roughly 30 million people, have chronic kidney disease (CKD) [1]. The prevalence of CKD is even higher in the adults 65–74 years, at one in five men, and one in four women [1]. Treatment for kidney failure costs 6.7% of the total Medicare budget to care for < 1% of the covered Medicare population [1]. CKD ranked 18th in the world in 2010 as the cause of death in the global burden of disease study [2]. Thus, CKD constitutes a significant public health burden.

Gout is an inflammatory arthritis characterized by hyperuricemia and monosodium crystal formation in joints and other tissue, which lead to local and systemic inflammation with up-regulation of cytokines such as interleukin-1 and others [3]. Systematic reviews and meta-analyses of observational studies linked hyperuricemia to the development of stage 3 chronic kidney disease (CKD) [4] and incident kidney disease defined as composite of CKD, end-stage renal disease (ESRD), albuminuria or elevated serum creatinine [5]. Animal studies support a role of hyperuricemia in the progression of renal disease [6]. A key unanswered question is whether gout increases the risk of new onset CKD and if so, does this risk vary by the key demographic and clinical characteristics.

## Methods

### Data sources and study sample

We used the 5% Medicare claims for this cohort study obtained through the Centers for Medicare and Medicaid Services Chronic Condition Data Warehouse. Patients were eligible for this study if they Medicare beneficiaries enrolled in Medicare fee-for-service (Parts A, B) 2006–2012, and not enrolled in Medicare Advantage Plan (due to incomplete claims data) and lived in the U.S. from 2006 to 2012. The study was approved by the Institutional Review Board at the University of Alabama at Birmingham.

### Independent variable/outcome of interest

The outcome of interest was incident chronic kidney disease (CKD)/kidney failure (referred to as CKD in this study), identified by the occurrence of two diagnoses for CKD at least 4-weeks apart in Medicare claims, identified with International Classification of Diseases, ninth revision, common modification (ICD-9-CM) codes, 582.xx, 583.xx, 585.xx, 586.xx or 588.xx, with an absence of any CKD code in the baseline 365-day period. This ICD-9-CM code based approach has been used to assess renal disease in the validated Charlson-Romano comorbidity index [7] (one of the most commonly used comorbidity index), and is being currently used by the U.S. Renal Data System Coordinating Center [8]; a similar set of codes is also used in Deyo-Charlson index [9], another adaptation of the Charlson index. This approach is valid with high specificity of 99% and moderate sensitivity of 70–88% [10] and a median positive predictive value of 78% [11]. These ICD-9 codes include all CKD stages of the National Kidney Foundation classification of CKD.

### Predictor of interest

Gout was the main exposure of interest, i.e., the independent variable. We assessed the presence of gout based on the presence of gout diagnosis identified with ICD-9 code of 274.xx in two Medicare claims at least 4-weeks apart, a valid approach with sensitivity of 90% and specificity of 100% [12].

We included known or suspected confounders/risk factors for CKD in our study, namely, demographics (age, gender and race), medical comorbidity assessed with a validated claims-based Charlson-Romano comorbidity index [7] (which includes diabetes, diabetes with complications, heart failure etc.), as well as hypertension, hyperlipidemia and coronary heart disease. We also included obesity as a covariate, realizing that it would be under-coded, considering the pros vs. cons. We also assessed for the use of cardiovascular drugs (statins, beta-blockers, diuretics, and angiotensin converting enzyme (ACE)-inhibitors), and gout drugs (allopurinol, febuxostat), as surrogate of cardiovascular disease and/or its severity, and/or their independent effects on pathways that contribute to inflammation and oxidative stress that can contribute to a potentially higher risk of CKD [13–15].

### Statistical analysis

We compared the characteristics of patients with to those without incident CKD, and calculated crude incidence rates of incident CKD in people with vs. without gout. We used multivariable-adjusted Cox regression analyses to assess the association of gout (exposure) with incident CKD (outcome), adjusting for demographics (age, gender, race), comorbidity (Charlson-Romano comorbidity index), and medications used for cardiovascular diseases or gout (main model; model 1). Hazard ratios and 95% confidence intervals (CI) were calculated. We conducted additional sensitivity analyses to test the robustness of findings by: (1) modeling Charlson-Romano comorbidity index as a categorical variable (model 2) or with individual comorbidities instead of continuous score plus hypertension, heart disease, obesity, and coronary artery disease (model 3); and (2) limiting to renal disease codes of ICD-9 codes 582.xx and 585.xx, more specific for CKD, and examining the effect on hazard ratios from models 1–3.

Subgroup analyses were done by age, gender, race as well as by the presence/absence of diabetes, hypertension, and coronary heart disease, known common risk factors for CKD in the general population.

## Results

Of the 1,699,613 eligible people, 168,065 developed incident CKD. Compared to people without gout, people with gout were 0.4 years younger, had higher medical comorbidity, hypertension, hyperlipidemia and coronary artery disease and were more likely to be male, African-American (Table 1).

**Table 1:** Demographic and clinical characteristics of people with vs. without Gout

	No Gout (*N* = 1,623,304)	Gout (*N* = 76,309)
Age, Mean (SD)	75.3 (7.6)	74.9 (7.0)
Gender, N (%)		
Male	672,755 (41.4%)	44,740 (58.6%)
Female	950,549 (58.6%)	31,569 (41.4%)
Race, N (%)		
White	1,403,749 (86.5%)	63,434 (83.1%)
Black	128,197 (7.9%)	8585 (11.3%)
Others	91,358 (5.6%)	4290 (5.6%)
Charlson-Romano comorbidity score^2^, Mean (SD)	1.5 (2.5)	2.0 (2.4)
Charlson-Romano comorbidity score, N (%)		
0	883,689 (54.4%)	28,407 (37.2%)
1	163,358 (10.1%)	10,658 (14.0%)
≥ 2	576,257 (35.5%)	37,246 (48.8%)
Select Charlson-Romano comorbidities		
Myocardial Infarction	59,625 (3.7%)	4450 (5.8%)
Heart Failure	172,489 (10.6%)	14,630 (19.2%)
Peripheral vascular disease	148,616 (9.2%)	10,082 (13.2%)
Cerebrovascular disease	152,599 (9.4%)	9046 (11.9%)
Dementia	74,277 (4.6%)	1926 (2.5%)
Chronic pulmonary disease	245,086 (15.1%)	15,297 (20.0%)
Connective tissue disease	43,359 (2.7%)	3063 (4.0%)
Peptic ulcer disease	29,277 (1.8%)	1923 (2.5%)
Mild liver disease	7583 (0.5%)	533 (0.7%)
Diabetes	280,819 (17.3%)	21,459 (28.1%)
Diabetes with end organ damage	77,421 (4.8%)	5906 (7.7%)
Hemiplegia	13,144 (0.8%)	634 (0.8%)
Hypertension	752,833 (46.4%)	53,555 (70.2%)
Hyperlipidemia	545,421 (33.6%)	37,898 (49.7%)
Coronary artery disease	267,429 (16.5%)	21,123 (27.7%)
Obesity	30,761 (1.9%)	3285 (4.3%)

Incident CKD developed in 150,162 people without gout and 17,903 people with gout, with crude respective incidence rates of 15.6 vs. 78.1 per 1000 person-years. Gout was associated with a higher hazard of incident CKD in multivariable-adjusted analyses, HR was 3.05 (95% CI, 2.99, 3.10), with minimal attenuation in sensitivity analyses, with HR 2.96 (95% CI, 2.91, 3.01) (model 2, categorical Charlson-Romano) and 2.59 (95% CI, 2.54, 2.63) (model 3, Charlson-Romano comorbidities plus hypertension, heart disease, obesity, and coronary artery disease) (Table 2). Of all comorbidities, diabetes and hypertension were associated with highest HR of 1.71 (95% CI, 1.69, 1.73) and 1.78 (95% CI, 1.76, 1.81) for incident CKD in multivariable-adjusted analyses, respectively.

**Table 2:** Association of gout and other risk factors with Incident CKD

	Multivariable-adjusted (Model 1)*		Multivariable-adjusted (Model 2)*		Multivariable-adjusted (Model 3)*	
	HR (95% CI)	*P*-value	HR (95% CI)	*P*-value	HR (95% CI)	*P*-value
Age (in years)						
65 - < 75	Ref		Ref		Ref	
75 - < 85	**1.62 (1.60, 1.63)**	**< 0.0001**	**1.60 (1.58, 1.62)**	**< 0.0001**	**1.55 (1.53, 1.57)**	**< 0.0001**
≥ 85	**2.26 (2.23, 2.30)**	**< 0.0001**	**2.27 (2.24, 2.31)**	**< 0.0001**	**2.24 (2.21, 2.27)**	**< 0.0001**
Gender						
Male	Ref		Ref		Ref	
Female	**0.78 (0.77, 0.79)**	**< 0.0001**	**0.78 (0.77, 0.78)**	**< 0.0001**	**0.76 (0.75, 0.76)**	**< 0.0001**
Race						
White	Ref		Ref		Ref	
Black	**1.38 (1.36, 1.40)**	**< 0.0001**	**1.42 (1.39, 1.44)**	**< 0.0001**	**1.30 (1.28, 1.32)**	**< 0.0001**
Other	**0.98 (0.96, 1.00)**	**0.024**	**1.01 (0.99, 1.03)**	**0.46**	**0.96 (0.94, 0.98)**	**< 0.0001**
Charlson-Romano score, per unit change	**1.22 (1.21, 1.22)**	**< 0.0001**	N/A		N/A	
Charlson-Romano score						
0	N/A		Ref		N/A	
1			**2.33 (2.30, 2.37)**	**< 0.0001**		
≥ 2			**2.99 (2.96, 3.02)**	**< 0.0001**		
Gout	**3.05 (2.99, 3.10)**	**< 0.0001**	**2.96 (2.91, 3.01)**	**< 0.0001**	**2.59 (2.54, 2.63)**	**< 0.0001**

* Model 1 included Charlson-Romano score as a continuous variable; Model 2 replaced it with categorized Charlson-Romano score; and Model 3 replaced it with each of the 17 Charlson-Romano comorbidities. All models were also adjusted for medications for cardiovascular diseases (statins, beta-blockers, diuretics, ACE-inhibitors) and for urate-lowering therapies for gout (allopurinol, febuxostat)

*N/A* not applicable, *HR* Hazard ratio, *CI* confidence interval, *Ref* referent category

Bold represents statistical significance, with a *P*-value < 0.05

Sensitivity analyses that limited the ICD-9 codes to more specific codes for CKD, confirmed findings from the three models with HRs of 3.10 (95% CI, 3.05, 3.15; Model 1), 3.03 (95% CI, 2.97, 3.08; Model 2) and 2.60 (95% CI, 2.56, 2.65; Model 3).

Subgroup analyses showed that association of gout with incident CKD differed statistically significantly by age, gender and race, interaction term *p*-values were < 0.0001, < 0.0001 and 0.004, respectively (Table 3).

**Table 3:** Association of gout with Incident CKD, in pre-defined subgroup analyses by race, gender, and age

	Multivariable-adjusted (Model 1)		Multivariable-adjusted (Model 1)		Multivariable-adjusted (Model 1)	
	HR (95% CI)	*P*-value	HR (95% CI)	*P*-value	HR (95% CI)	*P*-value
	Black		White		Other race	
Gout	**3.06 (2.93, 3.21)**	**< 0.0001**	**3.04 (2.98, 3.10)**	**< 0.0001**	**3.13 (2.91, 3.37)**	**< 0.0001**
	Female		Male			
Gout	**3.18 (3.09, 3.26)**	**< 0.0001**	**2.96 (2.90, 3.03)**	**< 0.0001**		
	65–75 years		75–85 years		> 85 years	
Gout	**3.34 (3.26, 3.43)**	**< 0.0001**	**2.87 (2.80, 2.95)**	**< 0.0001**	**2.66 (2.54, 2.80)**	**< 0.0001**
	No Hypertension		Hypertension			
Gout	**4.29 (4.15, 4.44)**	**< 0.0001**	**2.38 (2.33, 2.43)**	**< 0.0001**		
	No Diabetes		Diabetes			
Gout	**3.47 (3.40, 3.54)**	**< 0.0001**	**2.24 (2.18, 2.30)**	**< 0.0001**		
	No CAD		CAD			
Gout	**3.32 (3.25, 3.39)**	**< 0.0001**	**2.38 (2.31, 2.46)**	**< 0.0001**		

*P*-value for interactions were as follows

Race*gout interaction term, *p*-value 0.004

Sex*gout interaction term, *p*-value < 0.0001

Age*gout interaction term, *p*-value < 0.0001

Hypertension*gout interaction term, *p*-value < 0.0001

Diabetes*gout interaction term, *p*-value < 0.0001

CAD*gout interaction term, *p*-value < 0.0001

*HR* Hazard ratio, *CI* confidence interval

Hazard ratios that are significant with *p*-value < 0.05 are in bold

Subgroup analyses by key disease risk factor for incident CKD showed interesting findings and all interactions were statistically significant (< 0.0001). For example, in people without hypertension, diabetes or CAD, HRs of incident CKD with gout were, 4.29, 3.47 and 3.32, much higher than the respective HRs in people without each disease respectively, 2.38, 2.24 and 2.38 (Table 3).

## Discussion

We found that gout was associated with 3-fold higher hazard of CKD, independent of other risk factors including demographics, medical comorbidity including diabetes, hypertension and cardiovascular disease and the use of medications for cardiovascular disease and gout. An important finding was that the strength of association of gout with incident CKD changed minimally in multiple sensitivity analyses and only decreased from 3.1-fold in the main model with demographics, numerical Charlson-Romano comorbidities and commonly used medications to 2.6-fold in the model that additionally adjusted for hypertension, hyperlipidemia, obesity and coronary artery disease and examined individual Charlson-Romano comorbidities. This indicates that our study finding was robust, i.e., gout was independently associated with incident CKD in adults 65 years or older after controlling for common risk factors for CKD, including hypertension, diabetes, heart disease, obesity, age, race, gender and other medical comorbidities.

We can only speculate about possible mechanisms, since lack of laboratory data in Medicare and the observational nature study did not allow us to investigate this further in our study. Gout is characterized by the formation of monosodium urate (MSU) crystals, which are phagocytosed by macrophages or monocytes, which lead to disruption of lysosome and the activation of NALP3 inflammasome [16]. This process results in the formation of IL-1β and other pro-inflammatory cytokines [3]. Innate immune system is also involved in the pathogenesis of CKD, as demonstrated in the following observations. NALP3 mRNA levels were increased and correlated with renal function in a variety of nondiabetic kidney diseases and chronic kidney disease [13, 15]. Activation of NALP3 inflammasome and secretion of IL-1β and IL-18 causes the development of tubulointerstitial disease in diabetic nephropathy [17]. Severity of proteinuria in diabetic patients correlates with protein expression of caspase-1, IL-1β and IL-18 in the proximal and distal tubules [18]. Increased serum IL-18 levels in patients with chronic kidney disease [14] and the correlation of urine IL-18 levels with disease activity in nephrotic syndrome indicates that pro-inflammatory cytokines may play a role in chronic kidney disease [19, 20]. Therefore, a biological rationale exists for the association of gout with CKD. Future studies should assess whether interventions targeting these pathways can reduce the risk of CKD, slow down the progression of CKD or prevent CKD in the older adults with gout.

While we speculate that inflammation is one potential pathway that may underlie the association of gout with incident CKD, hyperuricemia might also contribute. Uric acid is primarily excreted as well by reabsorbed in the kidney. Urate may induce renal damage in its soluble (crystal-independent) or crystal form [21]. Soluble or crystalline urate can activate various pathways that lead to inflammatory, proliferative, and maladaptive changes in glomeruli (soluble urate only) and the tubulointerstitium [21]. Hyperuricemia has been considered to be a result of slowly progressive CKD [22, 23] by some, while others showed that hyperuricemia may be causative of CKD [24–28]. Allopurinol and febuxostat, two urate-lowering therapies (ULTs), have a potential role in preserving renal function. Evidence from observational studies indicated that use of allopurinol use was associated with improved renal function or slower age-related decline in renal function in some [29–31] but not all [32–35] studies. Similar evidence supported potential improvement of renal function with febuxostat use including an observational study [36] and a small randomized study [37]. A systematic review concluded that ULT with allopurinol may retard the progression of CKD, but that randomized trials were needed [38], a conclusion we agree with.

Another novel observation was the variation in the strength of the association of gout with incident CKD by important patient characteristics such as diabetes and CAD, and especially by hypertension. In each case, gout had a much stronger association with incident CKD in the absence of the condition: DM vs. no DM, 2.24 vs. 3.47; CAD vs. no CAD, 2.38 vs. 3.32; and hypertension vs. no hypertension, 2.38 vs. 4.29. These differences are not only statistically significant, but also clinically meaningful, considering that CKD is a common disease with a significant impact on patient morbidity and mortality. The HR of 4.3 of CKD with gout in the absence of hypertension is almost twice as much as the HR of 2.4 in the presence of hypertension. Much smaller interactions that were statistically significant were noted by age, gender and race. This is not surprising at all, since in the presence of hypertension, diabetes, heart disease, the pathogenic mechanisms of gout may play a smaller role in contributing to the risk of incident CKD. Regardless a hazard ratio of > 2 is an impressive finding of an association from a multivariable-adjusted model.

Study limitations include misclassification bias due to the use of diagnostic codes. We did not rely on a single diagnostic code, but used a validated algorithm for defining incident CKD to reduce this potential bias. This definition of incident CKD has been used in several high-quality studies [39–42] and being currently used by the U.S. Renal Data System Coordinating Center [8]. We realize that this ICD-9-code based approach includes all CKD stages of the National Kidney Foundation classification of CKD. Since milder kidney disease such as the CKD stage 2 likely has different outcomes than CKD stages 3–5, future studies need to investigate the risk of different stages of CKD in larger patient samples. Inclusion of CKD stage 2 in our ICD-9 approach may also lead to over-ascertainment of clinically significant CKD compared to the existing literature that relies on estimates GFR or creatinine. Some codes in our CKD definition are not specific, therefore we conducted sensitivity analyses limiting to codes specific for CKD, with minimal/no attenuation of the hazard ratios. We attempted to minimize confounding bias by including several confounders and known risk factors for chronic renal disease, including diabetes, hypertension, hyperlipidemia, heart disease, age, race, gender etc. [43, 44]. However, we were unable to control for other minor risk factors such as smoking, and genetics due to the absence of these data in Medicare data; and the use of non-steroidal anti-inflammatory drugs (NSAIDs) since the majority use in the U.S. is non-prescription, which is not available in the Medicare database. We did not control for the use of angiotension II receptor antagonists, which are nephroprotective. Medicare data does not include laboratory data, and therefore in-depth investigation of serum urate levels, or association with various stages of CKD based on estimated glomerular filtration rate could not be performed.

Study strengths include robustness of estimates in multiple sensitivity analyses, use of a representative sample of the U.S. elderly 65 years or older, and a large sample size.

## Conclusion

In conclusion, we found that the presence of gout was associated with a 3-fold higher risk of CKD, after controlling for demographics, comorbidity and cardiovascular and gout medication use. We also noted that this risk differed statistically significantly by age, gender and race, and both statistically significantly and clinically meaningfully by the presence of diabetes and CAD, and especially by hypertension. These observations improve our current understanding of renal disease in people with gout. Future studies need to elucidate the key pathogenic pathways responsible for reduced renal function in people with gout.

## Abbreviations

ACE: angiotensin converting enzyme
CAD: coronary artery disease
CI: confidence interval
CKD: chronic kidney disease
HR: Hazard ratio
ICD-9-CM: International Classification of Diseases, ninth revision, common modification
MSU: monosodium urate crystals
SD: standard deviation
